# Self-reported sleep disturbances in renal transplant recipients

**DOI:** 10.1186/1471-2369-14-220

**Published:** 2013-10-10

**Authors:** Hanna Burkhalter, Daniel P Brunner, Anna Wirz-Justice, Christian Cajochen, Terri E Weaver, Jürg Steiger, Thomas Fehr, Reto M Venzin, Sabina De Geest

**Affiliations:** 1 Institute of Nursing Science, University of Basel, Basel, Switzerland; 2 Centre for Sleep Medicine Hirslanden, Zürich, Switzerland; 3 Centre for Chronobiology, Psychiatric Clinics, University of Basel, Basel, Switzerland; 4 Department of Biobehavioral and Health Sciences, University of Illinois Chicago College of Nursing, Chicago, USA; 5 Division of Transplant Immunology and Nephrology, University Hospital Basel, Basel, Switzerland; 6 Division of Nephrology, University Hospital Zürich, Zürich, Switzerland; 7 Division of Nephrology, University Hospital Bern, Bern, Switzerland; 8 Center for Health Services and Nursing Research, KU Leuven, Belgium

**Keywords:** Renal transplantation, Sleep disturbances, Sleep quality, Daytime sleepiness

## Abstract

**Background:**

Poor sleep quality (SQ) and daytime sleepiness (DS) are common in renal transplant (RTx) recipients; however, related data are rare. This study describes the prevalence and frequency of self-reported sleep disturbances in RTx recipients.

**Methods:**

This cross-sectional study included 249 RTx recipients transplanted at three Swiss transplant centers. All had reported poor SQ and / or DS in a previous study. With the Survey of Sleep (SOS) self-report questionnaire, we screened for sleep and health habits, sleep history, main sleep problems and sleep-related disturbances. To determine a basis for preliminary sleep diagnoses according to the International Classification of Sleep Disorders (ICSD), 164 subjects were interviewed (48 in person, 116 via telephone and 85 refused). Descriptive statistics were used to analyze the data and to determine the frequencies and prevalences of specific sleep disorders.

**Results:**

The sample had a mean age of 59.1 ± 11.6 years (60.2% male); mean time since Tx was 11.1 ± 7.0 years. The most frequent sleep problem was difficulty staying asleep (49.4%), followed by problems falling asleep (32.1%). The most prevalent sleep disturbance was the need to urinate (62.9%), and 27% reported reduced daytime functionality. Interview data showed that most suffered from the first ICSD category: insomnias.

**Conclusion:**

Though often disregarded in RTx recipients, sleep is an essential factor of wellbeing. Our findings show high prevalences and incidences of insomnias, with negative impacts on daytime functionality. This indicates a need for further research on the clinical consequences of sleep disturbances and the benefits of insomnia treatment in RTx recipients.

## Background

Poor sleep quality is common among renal transplant (RTx) recipients, with a prevalence ranging from 30% to 62% [1-4] as measured using the Pittsburgh Sleep Quality Index (PSQI). Subjective sleep quality (SQ) is an evaluation of sleep by the affected individual [5], covering elements such as total sleep time, sleep onset latency, total waking time, sleep efficiency and disruptive events. Daytime sleepiness (DS) involves difficulty maintaining a desired level of wakefulness, i.e., the feeling of drowsiness with a tendency to doze [6].

One cross-sectional study using the PSQI in a Swiss transplant center reported a poor SQ prevalence of 47.4% [7]. As measured using the Epworth Sleepiness Scale (ESS), [8] data from three Swiss transplant centers showed a prevalence of 52% for poor SQ [8] and 50.9% for daytime sleepiness (DS). Most cross-sectional studies suggest that poor SQ is higher pre-RTx (49%-78% [3,9,10]) than post-RTx (30%-52% [1,11]). Similarly, insomnia (difficulty falling asleep, staying asleep, waking up before the desired time and being left tired during the day) in RTx candidates [12] has a prevalence of 15% in patients on maintenance dialysis, compared to 8% post-RTx [13]. Post-RTx SQ remains constant [14]. Supporting these findings, Sabbatini et al. (2005) showed that sleep significantly improved from pre- (PSQI mean: 8.52 ± 3.81, P < 0.001) to post-RTx (PSQI mean: 6.46 ± 3.71, P < 0.001), although it remained higher than in control subjects (3.54 ± 1.61, P < 0.0001) [3]. Finally, poor SQ has been linked to pre-RTx impaired health status [14,15], with post-RTx health status improving alongside SQ [13,16].

The most frequent sleep disorders among hemodialysis patients are conditioned insomnia (unconscious association of bedtime with negative feelings), obstructive or central sleep apnea (repeated cessation of breathing during sleep), restless leg syndrome (an irresistible urge to move the legs) and periodic limb movement disorder (involuntarily limb movements) [17]. In patients with end-stage renal disease, several uremic and non-uremic factors are thought to contribute to the pathogenesis of sleep disorders [17]. Sleep apnea appears to be related to displacement of fluids which destabilize the control of breathing and narrow the upper airway [18]. Restless leg syndrome and periodic limb movement disorders are correlated with anemia, iron deficiency, and peripheral and central nervous system abnormalities. Therefore, most such disorders improve post-RTx [18]. Excessive daytime sleepiness occurs in approximately 50% of patients with end-stage renal disease [19], the etiology of which appears related to both uremia and sleep fragmentation [19].

Self-report screening questionnaires to assess sleep problems are an efficient preliminary step in a sleep diagnostic process before the first assessment interview. However, screening survey data analyses cannot do more than generally categorize sleep complaints. A detailed diagnosis of a sleep disorder requires sleep experts and specific diagnostic tools. In sleep medicine, diagnosis follows the International Classification of Sleep Disorders (ICSD), which groups disorders into eight categories: (1) Insomnias; (2) Sleep Related Breathing Disorders; (3) Hypersomnias (excessive daytime sleepiness); (4) Circadian Rhythm Sleep Disorders; (5) Parasomnias (strange movements, behaviors, emotions, perceptions, and dreams during sleep); (6) Sleep Related Movement Disorders; (7) Isolated Symptoms, Apparent Normal Variants and Unresolved Issues; and (8) Other Sleep Disorders [20]. ICSD diagnosis guidelines require a clinical assessment interview and may specify diagnostic tools (e.g., polysomnography [21] or biophysiological measurement [22]) to differentiate sleep disorders from others with similar symptoms.

In summary, sleep quality normally improves after RTx; [3] however, a high proportion of the current study’s RTx recipients were still suffering from sleep disorders several years post-Tx [8]. Prior to this study, no self-reported data existed on sleep disturbances among (post-Tx) RTx recipients. Therefore, the aims of this study were:

1) to describe the frequency of self-reported sleep disturbances in RTx recipients screened with the Survey of Sleep (SOS); and

2) based on structured sleep assessment interviews, to measure the prevalence of sleep disorders in RTx recipients.

## Methods

### Design, setting and sample

This study used a sequential cross-sectional multicenter design with a sample of 249 adult home-dwelling RTx patients, all of whom were participating in a larger study on sleep and daytime sleepiness. The inclusion criteria were: (1) RTx took place at one of the three participating Swiss transplant centers, (2) a functioning renal graft at least 6 months post-Tx, (3) the ability to understand and read German, (4) 18 years of age or older, and (5) participation in the preceding study with poor SQ (PSQI >5 [23]) and/or DS (ESS > 6 for increased DS [24]) scores. Candidates were excluded if they were undergoing dialysis or had not signed the written informed consent form.

The stage sampling approach used was based on candidates’ PSQI and ESS scores, both of which were assessed as a part of the larger study [8]. The PSQI is a self-rated questionnaire consisting of 19 items, assessing a wide variety of factors related to sleep quality over a 1 month period, including estimates of sleep duration and latency, and of the frequency and severity of specific sleep-related problems. These 19 items are grouped into seven component scores, each weighted equally on a 0–3 scale. The seven component scores are then summed to yield a global PSQI score, which has a range of 0–21; higher scores indicate worse sleep quality. A cut-off of > 5 points is used to classify patients as having poor sleep quality [23]. The *ESS* is a validated eight-item questionnaire to measure a subject’s expectation of dozing (falling into a light sleep) in eight hypothetical situations. Dozing probability ratings range from 0 (no probability) to 3 (high probability). An ESS total score ≥ 6 indicates DS [25]. A score ≥ 10 indicates that a person tends to become very sleepy and should seek medical advice [25]. All 249 provided self-reported Survey of Sleep (SOS) data; a sub-sample (n = 164) additionally participated in a sleep assessment interview (83 declined participation).

### Variables and measurements

Age (in years), gender, years since transplantation, body mass index (kg/m^2^), creatinine (μmol/l), hemoglobin (g/l) and drugs (including sleep drugs) were retrieved from the participants’ hospital medical charts. Comorbidity data were also extracted from patients’ charts and categorized using the Charlson comorbidity index [26], which assigns various weights to specific conditions. Each of the 19 noted conditions was assigned a score of 1, 2, 3, or 6, depending on the associated mortality risk. For each patient the scores were summed to provide his or her overall comorbidity score [26]. Sleep quality and daytime sleepiness was extracted from the preceding study and categorized in three groups: 1) PSQI ≤ 5 (good SQ) & ESS ≥ 6 (DS); 2) PSQI > 5 (poor SQ) & ESS < 6 (no DS); 3) PSQI > 5 (poor SQ)& ESS ≥ 6 (DS).

### Survey of sleep (SOS)

The self-reported **Survey of Sleep** (SOS) questionnaire was developed at the University of Pittsburgh and translated into German by the second author. It is often used to report sleep symptoms in insomnia patients, [22] and studies often employ it as a preparatory step before carrying out sleep assessment interviews [27,28]. The questionnaire consists of 7 sections: (1) **sleep overview** (existence of problem(s) (yes/no), general sleep problem (main complaint); duration (less/more than 1 year), course (getting worse, same, better, irregular), and frequency of the sleep problem (once/month, several times/week, nightly)); (2) **sleep habits** (including bedtime, get-up time and sleep latency in hours and minutes, whether the subject sleeps better in another location (yes/no), regularity of bedtimes (yes/no); (3) **sleep disturbances** (sleep-related symptoms and a list of 45 potential disturbances); (4) **daytime function** (typical feelings on getting up (energetic, optimistic, refreshed, low energy, irritable, depressed, confused, anxious); nap behavior (intentional or unintentional naps, dreaming during the naps (yes/no), feeling more alert after the nap (yes/no), daytime function (sleepiness (not at all, slightly, moderately, extremely), accidents because of sleepiness (yes/no), fatigue (not at all, slightly, moderate, extremely), having to close eyes during the day to relax (yes/no), impaired daytime function (yes/no), most functional period of the day (early or late morning, afternoon or evening; night; no particular time), (5) **health habits** (use of sleeping drugs (Yes/No), caffeine (amount in cups), nicotine (number of cigarettes per day), alcohol use (glass unit per day), (6) **sleep history** (select the main complaint); and (7) **medical history** (diagnoses, drugs) [29].

The estimated time necessary to complete the SOS is 30 minutes. There is no sum scoring of the items and as of the time of writing no validity or reliability measures are available for it, as it was developed as a guide for an sleep assessment interview and not as a diagnostic tool [22]. The complete Survey of Sleep (SOS) questionnaire is available on request from the second author.

### Sleep assessment interview

Data from the SOS were used to prepare and structure the sleep assessment interview. All responses indicating possible sleep disturbances were addressed and elaborated on in the interview, which was structured to follow the 7 SOS sections, and lasted approximately one hour. The information generated by the interview helped to exclude some sleep disorders; however, as no follow-up visits took place and no further sleep diagnostic measurements or tools were used, the given diagnoses according to the ICSD criteria [20] should be regarded as preliminary.

The interviewer (first author) was trained to perform sleep assessment interviews by a certified sleep specialist and somnologist at the Hirslanden Sleep Disorders Center in Zollikon, Switzerland. This training included an overview of sleep disorders and of the techniques used to diagnose them. The second author checked a random sample of the completed interview transcripts and evaluated the comprehensive justification (to provide inter-rater reliability) of the preliminary sleep diagnoses. He also provided back-up assistance in view of resolving difficulties in assessment or categorization of sleep disorders.

### Data collection

**Figure 1:**
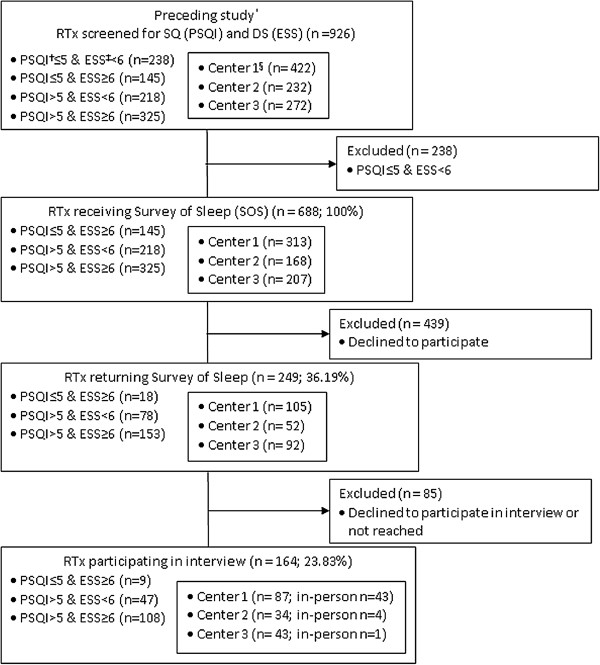
Flowchart of the sample.

Patients were informed at the start of the research project [8] that they might be invited for a further screening and assessment if their initial data indicated poor SQ and/or DS (see flowchart, Figure 1). Each such patient received a package containing an information letter, informed consent documents, a pre-stamped return envelope and the Survey of Sleep questionnaire (SOS). Candidates were included in the study if they signed the informed consent form, completed the SOS and returned the documents.

Data collection started in June 2011 at the first transplant center and ended in June 2012 at the third. Patients who had not responded within 2 months of the document mailings were contacted by phone to ask whether they had received the material and would still be willing to complete the questionnaire. Each eligible patient (N = 249) was contacted to set up a sleep assessment meeting, which could be conducted either in person or via telephone. Only 48 agreed to in-person interviews; 116 agreed to a phone interview. After 10 unsuccessful call attempts, the patients were categorized as unreachable or it was noted that they had declined to participate (n = 85). According to each participant’s wishes, the first author either met him/her at a predetermined place or called at the predetermined time.

The study was approved by the ethics committees of all three transplant centers (Ethikkommission beider Basel; Kantonale Ethikkommision Bern; Kantonale Ethikkommision Zürich). Data were anonymized following the interview and stored in an electronic databank. Participants given preliminary diagnoses were encouraged to consult their nephrologists regarding their sleep problems. Any patient who wished also received a list of certified sleep disorder centers in Switzerland for further examination and treatment.

### Statistical analysis

Descriptive statistics (means, standard deviations (SD), medians, quartiles, and frequencies) were used as appropriate, based on measurement levels and variable distributions. Likewise, comparisons between respondents and non-respondents were performed via t-test, Goodman and Kruskal's gamma test, or Mann–Whitney U test. Missing values were left blank and analysis was performed on the values given. SPSS® Statistics software (Version 19.0.0, IBM Corporation, Somers NY) was used for statistical analysis, with all critical probability levels set to 5%.

## Results

Of 688 RTx recipients invited to participate in this study, 249 (36.2%) agreed. Of 145 RTx with PSQI ≤ 5 (good SQ) & ESS ≥ 6 (excessive DS), 18 (12.41%) participated; of 218 with PSQI > 5 & ESS < 6, 78 (35.78%) participated; and of 325 with PSQI > 5 & ESS ≥ 6, 153 (47.08%) participated (Figure 1). Participants did not differ significantly from non-participants regarding age, gender, years since transplantation, comorbidities or daytime sleepiness. However, poor SQ (PSQI score >5) was significantly more prevalent among participants (Gamma: 479, df: 48; p = 0.0001). Of the 249 participants who filled in the SOS questionnaire, 164 (65.8%) participated in the subsequent sleep interview (Figure 1). The patients with PSQI > 5 (poor SQ) & ESS ≥ 6 (excessive DS) scores also had the highest participation rate in the assessment interview (65.8%). Most in-person sleep assessment interviews (n = 43) were performed with patients from center 1 in connection with a nephrology follow-up visit, where the first author has a clinical position. Participation in the sleep interview was much lower for patients in centers 2 and 3, as each interview required 1–4 hours of travel either for the patient or for the interviewer, and no possibilities existed to connect the interviews with nephrology follow-ups.

The participants had a mean age of 59.1 ± 11.6y; 60.2% were male and the mean time since RTx was 11.1 ± 7.0 years (Table 1). Immunosuppressive therapy, sleep drugs and co-medications, health habits and sleep history data are listed in Table 1. Sleep drug frequency, as noted in the nephrology charts, was 1.6% for benzodiazepines and 2.0% for other sleep drugs. The prevalence of self-reported sleep medication in the SOS was 32.9%.

**Table 1:** Characteristics of the sample [chart review and SOS part 5 (health habits), 6 (past sleep history) & 7 (medical history)]

**All (N = 249)**	***Frequency***	***Percentage***
Male	151	60.2
	*Mean*	*Std*
Age in years	59.6	12.1
Years since RTx	11.1	7.0
Body Mass Index (kg/m^2^)	25.9	5.2
Creatinine (μmol/l)	125.0	81.6
Haemoglobin (g/l)	127.6	16.5
	*Median*	*(25Q-75Q)*
Charlson Comorbidities Index	1	0-2
**Immunosuppressive drugs**	*Frequency*	*Percentage*
Cyclosporin	103	41.4
Tacrolimus	93	37.3
Sirolimus, Everolimus	23	9.2
Mycophenolat	152	61.0
Azathioprine	38	15.3
Corticosteroids	78	31.3
**Co-medication chart review**		
Statin	97	39.0
ACE inhibitor	61	24.5
Angiotensin receptor blocker	68	27.3
Calcium channel blocker	43	17.3
Beta blocker	92	36.9
Anticoagulants	58	23.3
Antidepressants	14	5.6
Diuretics	42	16.9
Sleep drugs	9	3.6
**Self-reported sleep drug use**	82	32.9
**Caffeine use** (>2 cups/d)	132	62.3
**Nicotine use**	31	12.4
**Alcohol use (**>1 glass/day)	64	25.8
**Sleep history in childhood**		
Insomnia	24	9.6
Sleepwalking	16	6.4
Bed-wetting	24	9.6
Talking in your sleep	29	11.6
Nightmares	21	8.4
Night terrors (screaming in the middle of the night and being difficult to awaken)	6	2.4
Head-banging or body rocking	8	3.2
Seizures during sleep, while falling asleep, or while waking up	5	2.0
Daytime sleepiness	12	4.8
Snoring	12	4.8
Breathing difficulties	5	2.0

### Prevalence and percentage of sleep problems and sleep habits [SOS part 1 & 2]

The most frequent sleep problem was difficulty staying asleep (49.4%), followed by difficulty falling asleep (32.1%) (Table 2). Most RTx recipients (61.4%) had experienced their sleep problems longer than 2 years without change (45%) and for 43.8% the problems occurred every night.

**Table 2:** General description of the sleep problem [SOS part 1 (overview) & 2 (sleep habits)]

**General sleep problem**	***Frequency***	***Percentage***
Having an actual problem with sleep or wakefulness	179	69.1
**Main sleep problem**		
Difficulty falling asleep	83	32.1
Difficulty staying asleep	128	49.4
Awakening early and being unable to fall back asleep	76	29.3
Excessive long sleep at night	21	8.1
Unusual behavior or experiences during sleep (e.g., nightmares, sleepwalking)	30	11.6
Excessive sleepiness during waking hours	66	25.5
Other problems	34	13.1
**Judgment of the sleep problem**		
Intense severity of the sleep problem (or problems)	67	26.9
Intense amount of interference with ability to function at home, at work, and with other people	48	19.3
Fairly intense sleepiness before bedtime	127	51.0
Better sleep outside compared to the sleep at home	11	4.4
Having regular sleep times	192	74.1
		
**Times related to sleep**	*Mean*	*Std*
Bedtime during the week	22.6	0.9
Bedtime at weekends	23.2	1.3
Time of lights off during the week	22.9	1.1
Sleep latency	28.1	19.3
Frequencies of sleep interruptions	2.8	1.8
Sleep latency after interruptions	21.9	16.4
Wakeup time during the week	6.5	1.2
Sleep duration	6.4	1.5
Get up time during the week	6.9	1.1
Get up time at weekends	8.0	1.1
	*Frequency*	*Percentage*
**How long having sleep problem**		
Between 6 months and 2 years	74	29.7
Between 2 and 5 years	60	24.1
>5 years	93	37.3
**Course of the problem**		
Becoming worse	15	6.0
Same	112	45.0
Improving	17	6.8
Irregular	79	31.7
Recurring regularly	8	3.2
**Frequency**		
Every day/night	109	43.8
Sometimes in a week	24	9.6
Sometimes in a month	31	12.4

### Prevalence and percentage of sleep symptoms [SOS part 3]

Of 45 sleep-related symptoms, the most prevalent were the need to urinate (62.9%), leg cramps during sleep (37.8%), frequent tossing and turning in bed (37.1%), feeling too hot or too cold (33.2%) and awakening for no particular reason (29.7%) (Table 3).

**Table 3:** The 32 most prevalent sleep disturbances out of 45 [SOS part 3 (sleep disturbances)]

**Sleep Disturbance of N = 249**	***Frequency***	***Percentage***
Need to urinate	163	62.9
Leg cramps during sleep	98	37.8
Frequent tossing and turning	96	37.1
Feeling too hot or too cold	86	33.2
Awaken for no particular reason (spontaneous awakenings)	77	29.7
Feeling anxious or emotionally tense, or worrying about things at bedtime	72	27.8
Physical nervousness and agitation in the evening or at night	68	26.2
Restless, uncomfortable, or “crawling” sensation in your legs during the evening or at night	62	23.9
Awakened by dreams (not nightmares)	56	21.6
Snoring	54	20.8
Feeling physically tense at bedtime	42	16.2
Awakening because of noise or light	38	14.7
Jerking or twitching in feet, legs, or arms during sleep	34	13.1
Large body jerks as you are falling asleep	33	12.7
Awakened by noises	32	12.4
Awakened by recurring dreams	31	12.0
Other pain during sleep	31	12.0
Muscle aches during or after sleep	30	11.6
Grinding teeth	26	10.0
Nightmares	26	10.0
Heartburns or other burning in chest, stomach	24	9.3
Headaches beginning during sleep	24	9.3
Palpitations, heart racing, or irregular heart beat	23	8.9
Other sleep disturbances	22	8.5
Talking in your sleep	19	7.3
Hallucinations as you are falling asleep or waking up, i.e., seeing or hearing things which turn out not to actually be real	15	5.8
Frequent cough	15	5.8
Episodes of confusion during sleep or upon awakening	14	5.4
Awakening choking, smothering, or gasping for air	13	5.0
Periods of not breathing during sleep	13	5.0
Difficulty breathing (including wheezing)	11	4.3
Difficulty swallowing	11	4.2

### Prevalence and percentage of daytime function [SOS part 4]

At wake-up time in the morning, 68 participants (26.2%) felt low energy, while an equal number felt optimistic. Only 16.9% napped unintentionally during the day; 47.2% napped intentionally. Half (49.8%) of all nappers felt more alert after a nap. During the day, 16.1% felt extreme sleepy, 16.9% intensely fatigued and 27.8% impaired in their daytime functions (Table 4).

**Table 4:** Description of daytime function [SOS part 4 (daytime function)]

	***Frequency***	***Percentage***
**Typical feelings at awakening in the morning**		
Optimistic	68	26.2
Low energy	68	26.2
Energetic	44	17.0
Refreshed	28	10.8
Irritable	18	6.9
Other	17	6.6
Depressed	16	6.2
Anxious	14	5.4
Confused	4	1.5
**Nap behavior**		
Intentional napping	118	47.4
Unintentional napping	42	16.9
Falling 1–2 times a day asleep or nap during the day	62	24.9
Often dreaming when falling asleep or nap during the day	10	3.9
Feeling more alert and awake after falling asleep or nap	124	49.8
**Daytime function**		
Extreme amount of sleepiness during daytime	40	16.1
Had an accident because of sleepiness or falling asleep	11	4.4
Intense amount of fatigue during the day	42	16.9
Have to close eyes during the day to relax	78	31.3
Impaired daytime functioning because of nighttime sleep disturbances, daytime sleepiness or fatigue	72	27.8
**Best function during the day**		
Early morning	102	39.4
Late morning	83	32.0
Early afternoon	38	14.7
Late afternoon	41	15.8
Early evening	34	13.1
Late evening	18	6.9
During the night	4	1.5
No specific time	35	13.5

### Prevalence and percentage of preliminary sleep diagnoses according to the ICSD

The most prevalent preliminary sleep diagnosis was chronic insomnia (42.5%), followed by circadian sleep-wake disturbances. Table 5 presents the preliminary sleep diagnoses based on a single assessment interview.

**Table 5:** Frequency of preliminary sleep diagnosis based on the interview grouped into the international classification of sleep disorders categories

**N = 164**	***Frequency***	***Percentage***
**Insomnias**		
Psychophysiological insomnia or paradoxical insomnia	53	32.3
Adjustment insomnia	3	1.8
Inadequate sleep hygiene	5	3.0
Insomnia due to medical condition	9	5.5
**Sleep Related Breathing Disorders**		
Obstructive Sleep Apnea, Adult	8	4.9
Other Sleep Related Breathing Disorders	5	3.0
**Hypersomnias of Central Origin Not Due to a Circadian Rhythm Sleep Disorder, Sleep Related Breathing Disorder or Other Cause of Disturbed Nocturnal Sleep**		
Behaviorally induced insufficient sleep syndrome	11	6.7
Idiopathic hypersomnia with long sleep time	7	4.3
Hypersomnia due to drug or substance use	1	0.6
**Circadian Rhythm Sleep Disorders (CRSD)**		
CRSD delayed sleep phase type	22	13.4
CRSD advanced sleep phase type	3	1.8
CRSD irregular Sleep-Wake Type	8	4.9
**Parasomnias**		
Nightmare Disorder	4	2.4
Parasomnia due to drug or substances	4	2.4
Confusional Arousals	4	2.4
Parasomnia due to med conditions	2	1.2
**Sleep Related Movement Disorders**	1	0.6
**Isolated Symptoms, Apparent Normal Variants, and Unresolved Issues**	0	0.0
**Other Sleep Disorders**	0	0.0
**No presumed diagnosis**	14	8.5

## Discussion

To our knowledge, this is the first study to focus on sleep problems in RTx recipients by using a detailed sleep questionnaire (SOS) and subsequent sleep assessment interview. This study describes the frequency of self-reported sleep disturbances in RTx recipients screened with the Survey of Sleep questionnaire (SOS) and the frequency of presumed sleep diagnoses based on the sleep interview.

As shown in Table 1, of the 688 patients invited to participate, roughly 50% (n = 325) registered poor SQ and DS. Figure 1 shows an increasing proportion of participants in the “poor SQ (PSQI > 5) & and DS (ESS ≥ 6)” group. Of these 325, 153 (47.1%) filled in the SOS and 108 (70.6%) participated in the assessment interview. In addition, poor SQ was significantly more prevalent in participants compared to non-participants. This would support a hypothesis that, even where no therapeutic benefit can be hoped for, patients are more likely to participate in studies directly relevant to their personal experience.

### Prevalence and percentages of sleep problems and sleep habits [SOS part 1]

The most prevalent sleep problem was difficulty staying asleep, followed by problems falling asleep. Both are characteristic of insomnia [20]. Other characteristics of insomnia common in this group were the extended duration of the sleep problem (61.4% reported durations greater than 2 years), the severity of the sleep problem (26.9% called their problems severe), the high prevalence of nightly sleeping pill use (32.9%), sleep latency of 28 ± 19.3 minutes, a high number of awakenings (2.8 ± 1.8) per night, long sleep latency after awakening (21.9 ± 16.4 minutes), and high ratios of time in bed to hours of sleep (8.3 ± 1.3 hours) to hours of sleep 6.4 ± 1.5. These results corroborate those of Moul et al. (2002), [30] who reported that 68% of insomnia patients exhibited long-term sleep problems (more than 1 year), severe sleep problems (81%), high nightly use of sleeping pills (89%), long sleep latency (53.3 ± 51.8 minutes), high numbers of awakenings (2.7 ± 1.7) per night, long sleep latency after awakening (56.0 ± 64.7 minutes), and high ratios of time in bed to hours of sleep (8.2 ± 1.9 hours in bed: 5 ± 1.7 hours of sleep). The average sleep duration of 6.4 ± 1.5 is very low, as studies have shown that chronic restriction of sleep to 6 h or less per night produces cognitive performance deficits equivalent to up to 2 nights of total sleep deprivation [31]. Sleep deficits seriously impair waking neurobehavioral functions (lapses in behavioral alertness) in healthy adults [31].

### Prevalence and percentages of sleep habits [SOS part 2]

One third of participants (n = 82) reported using sleeping pills; however, the medical chart data showed that very few (n = 9) had informed their nephrologists regarding their sleep problems or use of sleep medication. During their post-transplant hospitalization, all RTx recipients receive education regarding over-the-counter medication and medication prescriptions from other physicians, during which they are advised always to consult their nephrologist about possible interactions with their immunosuppressive drugs [32]. This discrepancy may indicate that patients are reluctant to bring up sleep problems, that they do not see sleep disorders as a topic that nephrologists can deal with, or that the nephrologists themselves simply consider sleep disorders a normal side effect of RTx immunosuppressive regimens. Compared to the general population, our prevalence of 32.9% self-reported sleep medication use is very plausible: sleep medications are used regularly by 3.2% of subjects 44 or younger, 13.3% of subjects between 45 and 64, 22% of those between 65 and 74 and 32% of individuals 75 or older [33].

### Prevalence and percentage of sleep symptoms [SOS part 3]

The most prevalent night-time symptom was nocturia. The frequency of its occurrence is key to further diagnosis. Nocturnal polyuria (nocturnal urine overproduction) and diminished nocturnal bladder capacity [34] require further testing to exclude urinary tract infections and prostate hyperplasia [35]. Also very prevalent were leg cramps and frequent turning in bed, indicating muscle fatigue, nerve dysfunction or electrolytic imbalances [36]. However, these symptoms could also be indices of restless leg syndrome, periodic limb movements, myositis, or peripheral neuropathy [36]. Similarly, turning or rocking in bed could indicate parasomnia (undesirable physical or behavioral phenomena occurring during the sleep period) [37]. For the diagnosis of parasomnias a careful physical examination is crucial and often a polysomnogram, including an expanded electroencephalographic montage, is necessary to distinguish between parasomnias (non-REM or REM) and nocturnal seizures [37].

Leg cramps during sleep were the second most prevalent sleep symptom (37.8%), followed by frequent tossing and turning in bed (37.1%). These two symptoms could be related to restless leg syndrome and/or periodic limb movements. The prevalence of restless leg syndrome in RTx recipients overall is 4.5% [38]. For periodic limb movements the overall prevalence is unknown, although there is an improvement from pre- to post-Tx [39]. Nocturnal leg cramps are often associated with vascular disease, lumbar canal stenosis, cirrhosis and hemodialysis [36], however no prevalence is known for RTx recipients. The sensorimotor symptoms of restless leg syndrome and/or periodic limb movements can be treated with dopamine agonists, gabapentin and its derivatives, and opioids [40]. To summarize, in-depth assessment of all these listed symptoms is crucial for the right treatment choice.

### Prevalence and percentage of daytime function [SOS part 4]

Table 4 shows the high prevalence of daytime sleepiness, tiredness and impaired daytime functioning, highlighting the importance for affected patients to use reminders (e.g., pillbox alarms, SMS reminder functions, or other cues) to ensure punctual intake of their immunosuppressive drugs. An earlier study showed correlations between DS and impaired immunosuppressive medication adherence [Burkhalter H, Wirz-Justice A, Cajochen C, Weaver T, Steiger J, Fehr T, Venzin R, De Geest S: Daytime sleepiness is associated with immunosuppressive non-adherence in renal transplant recipients: a cross-sectional multi-center study. Submitted]. However, it is possible that compensating behaviors such as increased use of mild stimulants (e.g., caffeine, nicotine) (Table 1) account for the lower prevalence of non-adherence (16%) than of DS (52%) [41].

Napping behavior and sleep duration depends on cultural, environmental, occupational and health factors [42]. In this study, 47.4% of participants reported intentional napping, a behavior shown to be protective against mortality [42]. However, a nap lasting several hours [43] might interfere with nighttime sleep–a point which would have to be borne in mind while counseling patients regarding sleep hygiene. The ideal nap duration for adults is about 10–20 minutes and the timing depends on the quality of sleep duration the preceding night, amount of prior wakefulness and morningness-eveningness tendencies [44].

### Prevalence and percentages of preliminary sleep diagnoses

This study’s most prevalent sleep diagnosis was chronic insomnia, followed by circadian rhythm sleep disorders. The prevalence of insomnia in the general population is 15-20% [45] and prevalence of circadian rhythm sleep disorders ranges from 3.1% in adults aged 40–64 to 16% in adolescents [46]. Our prevalence of 42.6% insomnia and 20.1% CRSD is only partially comparable based on our group’s pre-selection criteria (RTx recipients having poor SQ and/or DS). Various publications suggest RTx recipients’ sleep disorders are related to medications (e.g., β-blockers [47], nonsteroidal anti-inflammatory drugs [48], corticosteroids [49] and mycophenolic acid [50]) and other clinical conditions [51,52]. Molnar et al. [53] list numerous potential causes of sleep disorders in this group, including pre-existing sleep disorders, transplant surgery, hospitalization, anxiety and uncertainty, fear of organ rejection, immunosuppressive medication, deteriorating kidney function and co-morbid medical conditions, psychosocial problems, psychiatric and neurological disturbances, lifestyle, diet, environmental factors and aging. With so many possible contributing factors, the most appropriate course of action might be a referral to a sleep expert, who could counsel the patient on the full range of behavioral and medical interventions available, and help them to choose those best suited to their needs [54]. Sleep interventions for RTx recipients are the same as for the general population, apart from the risk of interaction with immunosuppressive therapy and the need to consider the long-term side effects of their therapy (e.g., osteoporosis, new onset of diabetes, pain).

### Limitation of this study

Since only 249 RTx recipients filled in the questionnaire, of which only 164 (65.9%) gave interviews, the generalizability of this study’s findings are limited. In addition, the high prevalence of RTx recipients in the “poor SQ (PSQI > 5) & and excessive DS (ESS ≥ 6)” group showing an increasing proportion along the study steps, limits the significance and comparability of the presumed sleep diagnoses.

### Suggested further research

Further research will be necessary to develop safe interventions for RTx recipients with sleep-wake disturbances, taking into account their impaired renal function (limited organ survival), high risk of skin cancer (a side-effect of immunosuppressive treatment) and need to adhere to their medication regimens (high risk of acute graft rejection). These interventions should include education [55] regarding sleep disorders and their negative health impacts. Apart from established cognitive and behavioral interventions for insomnia, new chronotherapeutics treatments, particularly bright light therapy and melatonin supplementation [56] should be investigated. For RTx recipients, who already have a high number of medications to ingest daily, light therapy might be a realistic intervention to stabilize sleep-wake rhythms compared to melatonin supplementation (one more drug to ingest).

## Conclusion

Our findings show high prevalence of insomnia and of impaired daytime functionality. This indicates a need for further research on the clinical consequences of sleep-wake disturbances and the benefits of insomnia treatment in RTx recipients.

## Abbreviations

CRSD: Circadian Rhythm Sleep Disorders; DS: Daytime sleepiness; ESS: Epworth Sleepiness Scale; ICSD: International Classification of Sleep Disorders; PSQI: Pittsburgh Sleep Quality Index; RTx: Renal transplant; SOS: Survey of sleep; SQ: Sleep quality.

## Competing interests

The results presented in this paper have not been published previously.

This study was funded by a research grant from the Swiss Renal Foundation (the Alfred and Erika Bär-Spycher Foundation). There are no conflicts of interest.

## Authors’ contributions

HB conceived this study with SDG, DPB, AWJ, JS, TF, RMV and TW. HB also coordinated the data collection with the three centers, and collected the data with the centers' physicians, JS, TF and RMV. HB performed the sleep assessments with the expert guidance of DPB. HB, DPB and SDG drafted the article and all authors read and approved the final manuscript.

## Pre-publication history

The pre-publication history for this paper can be accessed here:

http://www.biomedcentral.com/1471-2369/14/220/prepub
